# An ultra-fast mechanically active cell culture substrate

**DOI:** 10.1038/s41598-018-27915-y

**Published:** 2018-07-02

**Authors:** Alexandre Poulin, Matthias Imboden, Francesca Sorba, Serge Grazioli, Cristina Martin-Olmos, Samuel Rosset, Herbert Shea

**Affiliations:** 1 Institute of Microengineering, École Polytechnique Fédérale de Lausanne, CH-2002 Neuchâtel, Switzerland; 2 Swiss Center for Electronics and Microtechnologies (CSEM), CH-2002 Neuchâtel, Switzerland; 3 Division of Neonatology and Pediatric Intensive Care Medicine, University Hospital of Geneva, CH-1211 Geneva, Switzerland; 4 Department of Microbiology and Molecular Medicine, Faculty of Medicine, University of Geneva, CH-1211 Geneva, Switzerland; 5 Bioengineering Institute, University of Auckland, 1010 Auckland, New Zealand

## Abstract

We present a mechanically active cell culture substrate that produces complex strain patterns and generates extremely high strain rates. The transparent miniaturized cell stretcher is compatible with live cell microscopy and provides a very compact and portable alternative to other systems. A cell monolayer is cultured on a dielectric elastomer actuator (DEA) made of a 30 μm thick silicone membrane sandwiched between stretchable electrodes. A potential difference of several kV’s is applied across the electrodes to generate electrostatic forces and induce mechanical deformation of the silicone membrane. The DEA cell stretcher we present here applies up to 38% tensile and 12% compressive strain, while allowing real-time live cell imaging. It reaches the set strain in well under 1 ms and generates strain rates as high as 870 s^−1^, or 87%/ms. With the unique capability to stretch and compress cells, our ultra-fast device can reproduce the rich mechanical environment experienced by cells in normal physiological conditions, as well as in extreme conditions such as blunt force trauma. This new tool will help solving lingering questions in the field of mechanobiology, including the strain-rate dependence of axonal injury and the role of mechanics in actin stress fiber kinetics.

## Introduction

**Figure 1:**
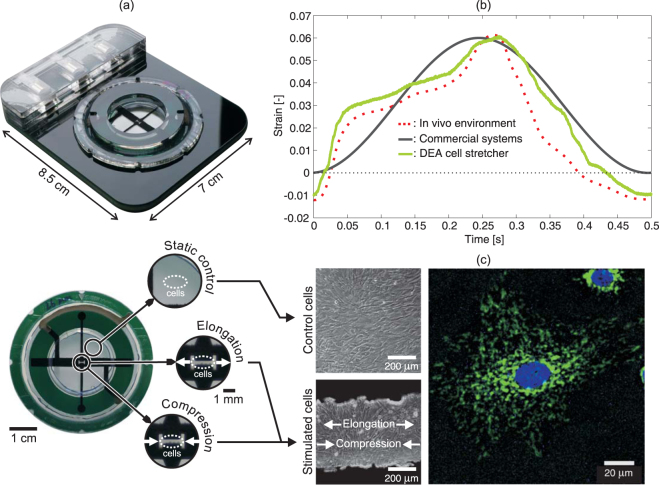
(**a**) Isometric view of the DEA-based cell stretcher placed in a compact holder that allows for simple connection to the voltage source. The bottom picture is a top view of the device. Electrodes are black, the elastomer is transparent and the frame is green. DEAs are patterned around a central region, in which the cells are cultured. By actuating the DEA electrodes, uniaxial tensile and compressive strain can be generated in the culture region. Cells cultured in the region between the DEA and the frame serve as a static control. (**b**) Plot of culture strain vs. time for three cases: the green trace is a measured complex strain-time profile generated with our device, reproducing the strain experienced by cells in the mitral valve of the human heart^42^, shown in red. The high strains and high strain rates generated by our device enable accurately reproducing the complex motion encountered *in vivo*. In commercial cell stretching systems, the strain-time profile is typically approximated as simple sinusoid as plotted in black. (**c**) Left: images of the static and active areas of the membrane with a monolayer of human bronchial smooth muscle cells cultured on the device. Right: human lung carcinoma cells grown on the device, imaged in fluorescence at 40x magnification, with DNA and mitochondria stained in blue and green, showing the high-quality imaging through the device.

The human body is constantly exposed to a complex and changing set of mechanical forces. The origin can be internal, e.g. muscle contraction and tissue growth, or external, e.g. impact force or gravity. Research in mechanobiology have shown that mechanical stimulation can influence fundamental cellular functions such as cell migration^1^, proliferation^2^ and gene expression^3^. Advances in this emerging field could lead to better diagnosis and treatment of common and serious medical conditions such as cardiovascular disease and cancer^4^. We present a fast electroactive silicone elastomer membrane for periodically stretching cell monolayers. The device is shown in Fig. 1(a). The sub-millisecond response time enables the application of precise and complex strain-time profiles, while the compact size and transparency enable live cell imaging during stretching, in both compression and tension. The device serves as a novel research tool in mechanobiology, a new field of science that studies how cells and tissues are affected by their mechanical environment^5,6^.

Mechanosensitivity is generally studied on cells cultured on a soft deformable membrane, that is periodically or statically stretched^7^. Extracellular matrix proteins such as fibronectin and collagen are used to promote cell adhesion, ensuring that cells deform with the membrane^8^. To mimic the *in vivo* mechanical environment, strain between 5% and 20% are typically studied at frequencies below 5 Hz and for durations ranging from 2 h to 48 h^6^. Several commercial systems^9,10^ are available, providing a reliable method to mechanically stimulate cultured cells at up to several Hz and strain rate of ~1 s^−1^. For applications where faster mechanical loading is required, strain rates equal to ~10 s^−1^ were reported for pneumatic actuators^11,12^, and strain rates equal to ~100 s^−1^ were reported for voice coil actuators^13,14^. The systems’ large size is however a major drawback in terms of compatibility with standard laboratory equipment such as incubators and optical microscopes. The size and cost makes high-throughput studies impractical, hindering statistical and parametric studies.

The limitations of current technologies and the growing interest in cell mechanobiology are driving the development of miniaturized cell stretchers^15^. While several actuation technologies have been reported^16–19^, the great majority of devices are based on microfluidics, using fluid pressure to deform a suspended culture membrane. Radial strain can be generated with a chamber located either below^20^ or around a circular membrane^21^. Linear strain can be generated by using a set of chambers located on opposite sides of a square membrane^22^. Greater control over the strain profile can be achieved by adding a second set of chambers^23^. These cell stretchers themselves are compact, but as they rely on pneumatic actuation, they require a connection to bulky external pressure or vacuum supplies, typically limiting their speed to several Hz, i.e. strain rates are limited to ~1 s^−1^. There is however a need for faster systems: strain rates in the order of 10 s^−1^ have be measured in the mitral valve^24^, and strain rates above 100 s^−1^ can be experienced during violent impacts leading to traumatic brain injuries^13,25^.

An alternative to pneumatic actuation is dielectric elastomer actuators (DEAs)^26,27^, a soft and compact actuation technology with large strain (>400%)^28,29^, fast response time (<1 ms)^30^, that can be miniaturized^31,32^ and integrated into arrays of individually addressable elements^33^. In its simplest configuration, a DEA consists of a soft (~1 MPa) dielectric elastomer membrane sandwiched between two stretchable electrodes^34^. The membranes are typically 10–100 μm-thick. When a voltage difference of several kV’s is applied between the electrodes, the membrane thickness decreases due to the applied electrostatic force, and the DEA surface area correspondingly expands due to the incompressibility of the elastomer. A variety of high force DEA configurations have been demontrated^35–38^. For stretching cells monolayers however, the smaller forces generated by single-layer in-plane devices are sufficient. For this configuration, the elastomer membrane is typically prestretched and bonded to a rigid frame, taking advantage of the elastomer’s hyperelastic properties to suppress electromechanical instability^39^ and to increase strain by using the region of the elastomer without electrodes as a spring.

We recently reported a DEA-based cell culture system and used it to apply periodic strain of 10% at 0.1 Hz to lymphatic endothelial cells, observing that the cells orient themselves perpendicular to the elongation direction^40^. This work showed that biocompatibility and possible issues related to stray electric fields when culturing cells on DEAs have been successfully addressed. The electric field induced by the high-voltages did not affect cell viability and development, as they were suitably shielded by the careful electrode design. A different group also reported a DEA that can generate radial strain to mimic the intestinal peristalsis^41^. While those two earlier studies focussed on showing compatibility of DEAs with living cells, here we report vastly improved actuation performance and compatibly with live imaging during stretching.

**Figure 2:**
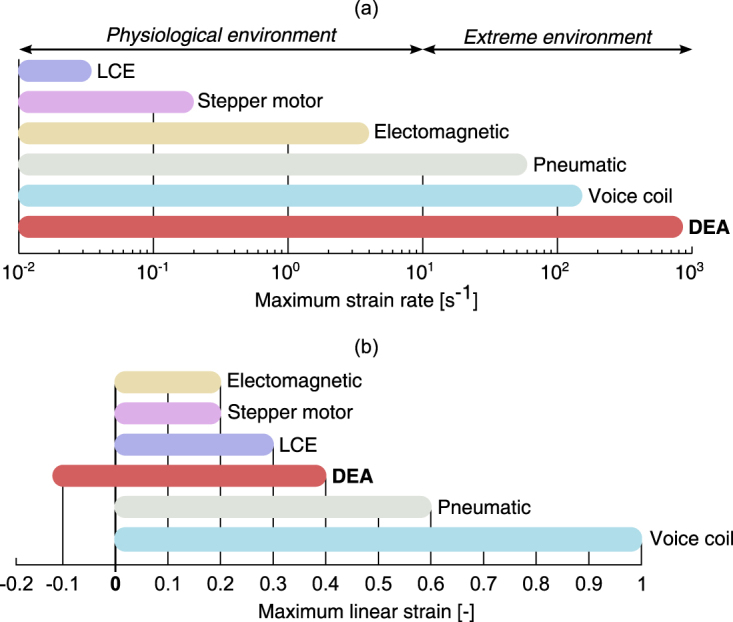
Reported performance of deformable cell culture substrate technologies in terms of maximum strain and strain rate. We limited the comparison to systems that generate planar deformation of the culture substrate. (**a**) The highest strain rate is achieved by our DEA cell stretcher (870 s^−1^). Systems using voice coil (150 s^−1^)^13^ and pneumatic (60 s^−1^)^22^ actuators can also generate extreme strain rates, whereas liquid crystal elastomer (LCE) actuators (0.035 s^−1^)^54^, stepper motor (0.2 s^−1^)^16^ and electromagnetic actuators (4 s^−1^)^55^ are limited to physiological strain rates. Piezoelectric devices^18^ have the potential to generate extreme strain rates but no values have been reported yet. (**b**) The DEA cell stretcher is the only system that showed mechanical stimulation profiles that combine tensile and compressive strain. All the reported technologies enables strain of 0.2 or greater, and the maximum reported strain of 1 was achieved using voice coil actuators^13^.

We present a DEA cell stretcher which can generate complex strain-time profile and precisely mimic the *in vivo* environment as illustrated in Fig. 1(b). The device we present here generates 38% tensile uniaxial strain, 12% compressive strain, provides sub-millisecond response time, and is compatible with fluorescence live cell imaging during stretching. Figure 2 compares the performance of our DEA cell stretcher with other deformable cell culture substrate technologies in terms of maximum strain and strain rate. We limited the comparison to systems that generate planar deformation of the culture substrate. The comparison charts show that our device can generate the highest strain rates, and is the only system for which mechanical stimulation combining tensile and compressive strain has been demonstrated.

The unique capabilities provided by the DEA cell stretcher enable new experiments that can foster progress in mechanobiology. To be biologically relevant, it is important to stimulate the cells with the rich dynamics they experience *in vivo*. Cell stretching systems are indeed often not capable of generating both tensile and compressive strain. The green line in Fig. 1(b) corresponds to a measured strain-time profile generated using our device that matches the mechanical environment measured in the human mitral valve^42^ (red line). Commercial systems typically generate the much simpler sinusoidal profile as shown in black. While the frequency of biologically relevant processes rarely exceed 10 Hz, the periodic strain-time profile can often include higher frequency components such as the ramp-to-plateau transition observed on Fig. 1(b), requiring ms scale time response to faithfully reproduce the strain-time profile. In extreme cases, such as traumatic brain injuries, full strain occurs in a few milliseconds^43^, which available techniques cannot reproduce. The DEA cell stretcher provides a new tool which can generate extreme mechanical environment and help solving important questions such as the effect of strain rate on axonal injury.

## Results and Discussion

### Challenges of interfacing DEAs with cells

Using DEAs as platforms for cell mechanobiology presents unique design and materials challenges because the dielectric elastomer membrane serves both as a soft transparent actuator and as cell culture substrate. The DEA (i.e., the elastomer, electrodes, electrical contacts, frame) must be cytocompatible and withstand routine cell culture protocols such as sterilization by immersion in ethanol, autoclaving or exposure to ultraviolet light. To be compatible with optical microscopy during stretching, the membrane must exhibit high transparency and low diffusion of visible light. We therefore chose a silicone elastomer (Sylgard 186, Dow Corning) as the membrane as it is one of the few elastomers that simultaneously meets the requirements for DEA speed, actuation strain, and optical transparency without intrinsic fluorescence. While acrylic elastomers such as VHB tape (3 M, USA) can provide larger actuation strain^44^, silicone elastomers allow for much longer lifetime as well as order of magnitude faster response due to their low viscoelasticity^30^. Silicone precursors are readily available in their liquid form, thus enabling full control on the membrane thickness. Reliably operating DEAs in conductive liquids is a challenge^45^. Our device is capable of stable operation during 24 h and tens of thousands of cycles while immersed in growth medium^40,46^ (see Supplementary Note 6). As described in the Materials and methods section, we encapsulate the bottom of the DEA in a 125 μm thick oil layer that allows long-term high-resolution imaging through the device (Fig. 1(c)), as well as preventing electrical breakdown as discussed below.

Depending on device geometry, the DEA electrodes may also be in contact with the cell culture. Carbon-grease or carbon-powder electrodes are not suitable materials since they would not withstand most sterilization protocols and contaminate the culture medium. Structurally stable electrodes^47^ such as carbon-elastomer composite^48^ or implanted gold electrodes^49^ are required for cell stretching applications. We selected a carbon-elastomer composite^50^ that was pad-printed and cured on the silicone membrane to create robust and cytocompatible electrodes with minimal stiffening impact. The growth medium is a conductive solution and therefore acts as a blanket electrode on top of the DEA. In addition to influencing DEA performance by changing electric field distribution^45^, ions from the growth medium can diffuse into the silicone membrane, driven by the applied electric field, changing its mechanical and electrical properties, leading to premature dielectric breakdown. In our design, the high-voltage electrodes align with opposing ground electrodes, hence limiting the fringing electric fields. With this optimized configuration, our prior work showed that the device fringing field has no effect on cell viability and on different cellular responses^40^. Should the fringing electric field be problematic for some types of cells, a slightly different design using three electrodes could be used to completely suppress the fringing electric field^51^.

### DEA design for both tensile and compressive strain

**Figure 3:**
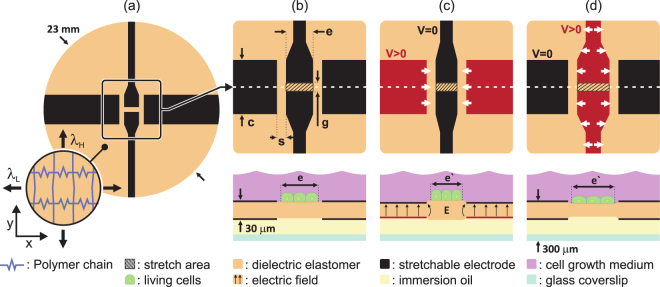
(**a**) The actuator consists of an elastomer membrane with four stretchable electrodes patterned on each side. The membrane is non-equibiaxially prestretched to suppress the electromechanical instability in the DEA and to set the preferred actuation direction. The elastomer is much stiffer in the y-axis than the x-axis due to the hyperelastic behavior of the silicone elastomer. (**b**) Cells are cultured at the surface of the device and immersed in growth medium. The bottom side is covered by a thin oil film and sealed with a glass coverslip. Upon actuation, precise strain can be generated on cells located at the center of the device (hatched rectangle). (**c**) When up to 4 kV are applied between the top and bottom horizontal electrodes (in red), the electrodes expands horizontally, hence compressing the cells in the center of the membrane. (**d**) When up to 4 kV are applied between the top and bottom vertical electrodes (in red) they expand horizontally, hence elongating cells located at the center.

While previously reported DEAs could only generate either tensile or compressive strain, the device presented here can both stretch and compress a given region. Figure 3(a) presents a schematic top view and cross-sections of the DEA cell stretcher. The active layer consists of an elastomer membrane with stretchable electrodes patterned on both sides. Note there are 4 top and 4 bottom electrodes. When a voltage difference is applied between electrodes located on both sides of the membrane, electrostatic forces squeeze the membrane, which as a result decreases in thickness and expands in area^34^.

Generating tensile and compressive uniaxial strain presents many challenges which were addressed by optimized choice of the non-equibiaxial membrane prestretch and of a 4 electrodes geometry. Cells are cultured directly on the region at the center of actuator and can be stretched or compressed upon actuation of the horizontal or vertical electrodes respectively. Figure 3 shows the operating principle, in which highly anisotropic prestretch is used to generate uniaxial strain. The DEA membrane is highly stretched by *λ*_*H*_ on the y-axis, and only slightly prestretched in the x-axis by *λ*_*L*_. The elastomers show a hyperelastic behavior, with strong increase in effective Young’s modulus as they are extended. On the y-axis, the polymer chains are prestretched nearly to their maximum extension. On the x-axis however, the chains can still easily be stretched further, and the effective membrane stiffness is thus much lower in the x-axis. The DEA then deforms preferentially along the x-axis^52^, converting the electrostatic forces into uniaxial planar motion. The maximum strain in our device is limited by loss of mechanical tension in the membrane (not by the electromechanical instabiltiy or diecletric brekadown). *λ*_*L*_ is an important parameter as it sets both the maximum strain and the strain anisotropy. Prestretch of *λ*_*H*_ = 2.7 and *λ*_*L*_ = 1.2 are applied on the DEA cell stretcher as described in the Materials and methods section.

The four stretchable electrodes located on the culture (top) side are connected to ground, whereas the four electrodes located on the bottom side are connected to high-voltage for actuation. The device is designed to generate a uniform strain profile on cells located in the hatched area at the center of the membrane, see Fig. 3(b). When the horizontal electrodes are actuated, they expand preferentially along the x-axis (i.e. orientation of lower prestretch), thus compressing the cells along the x-axis as shown in Fig. 3(c). When the vertical electrodes are actuated, their expansion preferentially stretches the cells along the x-axis (Fig. 3(d)).

The DEA strain field depends on the electrodes geometry. As shown in Fig. 3(b), the vertical electrodes are *e* = 1.5 mm wide and are separated by *g* = 0.5 mm. The horizontal electrodes are *c* = 3 mm wide and are separated from the vertical electrodes by *s* = 0.5 mm. The uniformity of the strain profile is mainly influenced by the aspect ratio *e*/*g*, for which higher values provide better strain uniformity. During actuation, up to 4 kV are applied across the *s* = 0.5 mm that separates the horizontal and vertical electrodes. The method for designing the electrode shapes and sizes is presented in Supplementary Note 1. To avoid electrical arcing between the two sets of bottom electrode, a 125 μm thick microfluidic reservoir is created on the bottom side of the membrane and filled with oil which exhibit higher dielectric strength than air.

### Uniaxial compression and elongation measurements

**Figure 4:**
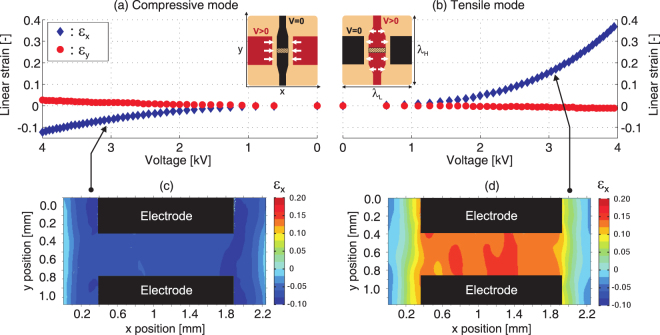
Linear strain as a function of driving voltage (**a**) in compressive mode using the horizontal electrodes and (**b**) in tensile mode using the vertical electrodes. The strain along the x-axis *ε*_*x*_ (red circles) and the y-axis *ε*_*y*_ (blue diamonds) were measured by tracking the boundaries of the central gap identified with a hatched rectangle. Most of the deformation occurs along the x-axis due to the high membrane prestretch applied along the y-axis (see Fig. 3(a)). The local strain mapping for *ε*_*x*_ was obtained using digital image correlation. In compressive mode, the device reaches −12% uniaxial strain with a (**c**) uniform strain field in the center of the membrane. In tensile mode, the device reaches 38% uniaxial strain and a (**d**) uniform strain field in the center of the membrane.

The device strain-voltage response as well as strain map at one voltage were measured in both tensile and compressive modes, as reported in Fig. 4.

The strain data in compression mode are presented in Fig. 4(a). In this configuration, only the horizontal electrodes on the bottom side of the membrane are at high-voltage. All top electrodes and the bottom vertical electrodes are grounded. The average strain was measured by tracking the four corners of the passive region shown as a hatched rectangle in the figure. Compressive strain along the x-axis of *ε*_*x*_ = −12.3% is reached at 4 kV, which corresponds to an electric field of 138 V μm^−1^ between the top and bottom electrodes. Due to the highly anisotropic prestretch of the membrane (see Fig. 3(a)), the compression along the x-axis is accompanied by only a small elongation along the y-axis of *ε*_*y*_ = 2.7%. The *ε*_*x*_/*ε*_*y*_ ratio provides a measure of how uniaxial the actuation is. The generated strain is the most uniaxial at 4 kV where *ε*_*x*_/*ε*_*y*_ = −4.6. Using digital image correlation, we determined the local strain profile from pictures of the device with cultured cells at rest and under actuation. The strain map is presented in Fig. 4(c). It shows very good uniformity in the center of the electrode gap with slightly higher strain at the left and right edges. The strain quickly drops to zero outside of the electrode gap, as expected.

The strain data in tensile mode are presented in Fig. 4(b). In this configuration, only the vertical electrodes on the bottom side of the membrane are connected to high-voltage. All top electrodes and the bottom horizontal electrodes are grounded. Tensile strain along the x-axis equal to *ε*_*x*_ = 38.3% was seen at 4 kV, which corresponds to an electric field of approximately 184 V μm^−1^. Due to the highly anisotropic prestretch of the membrane (see Fig. 3(a)), the elongation along the x-axis is accompanied by only a small compression along the y-axis equal to *ε*_*y*_ = −1.2%. The generated strain is the most uniaxial at 4 kV where *ε*_*x*_/*ε*_*y*_ = −31.9. The local strain map is presented in Fig. 4(d). It shows good uniformity over most of the electrode gap with slightly lower strain at the left and right edges. Similar to the compression case, the strain quickly drops to zero outside of the electrode gap. We thus can apply a uniform and continulously tunable strain from −12% to 38% to the cell culture region.

### Sub-millisecond response time

**Figure 5:**
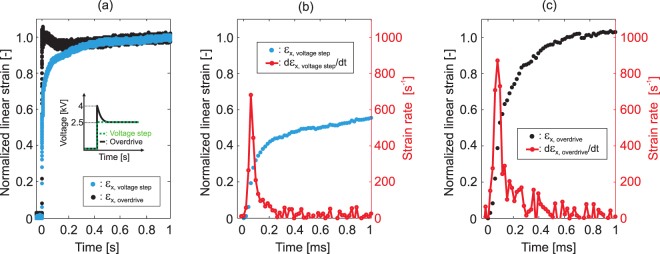
(**a**) Normalized linear strain along the x-axis *ε*_*x*_ as a function of time in response to a 2.5 kV voltage step (blue curve) and a 4 kV peak value overdrive waveform (black curve). The strain is normalized to the quasi-static value reached after 1 s of actuation. (**b**) In response to the 2.5 kV voltage step, the device reaches 50% of its maximum strain after 0.5 ms with a peak strain rate of 680 s^−1^. (**c**) In response to the overdrive waveform, the device reaches 50% of its maximum strain after 0.09 ms with a peak strain rate of 870 s^−1^.

One important advantage of DEAs is their very fast response time, when suitable membranes and electrodes with low viscoelasticity are used. The high-speed response of our device makes it possible to reproduce complex *in vivo* strain-time profiles as illustrated in Fig. 1, as well as reproduce extreme mechanical environments to study injuries caused by violent impact such as blunt trauma. Figure 5 plots the response time of the cell stretcher in tensile mode. The driving signal was generated using a programmable waveform generator connected to a high-voltage power amplifier. A high-speed camera was used to record the device. The actuation strain was determined by tracking the boundaries of the electrode gap as described in the Materials and methods section.

The device mechanical strain response to a 2.5 kV voltage step is presented in Fig. 5(a). The blue line corresponds to the normalized actuation strain *ε*_*x*_ as a function of time *t*. The voltage is applied at *t* = 0 s. The strain is normalized to the value measured at *t* = 1 s where the device has reached a quasi-static position. Data for a 2.5 kV drive signal is presented here, and nearly identical response was observed for voltages up to 4 kV (see Supplementary Note 3 for data at the higher voltages). The device response in the first 1 ms is presented in Fig. 5(b), with the normalized strain in blue and the corresponding strain rate in red. The device reaches 50% of the maximum strain in 0.5 ms, generating strain rates as high as 680 s^−1^. When driven with a 3.5 kV sinusoidal signal, the cutoff frequency of the system is equal to 800 Hz (see Supplementary Note 5).

The device response to a custom signal labeled “overdrive” is presented Fig. 5(a). The black line corresponds to the normalized actuation strain *ε*_*x*_ as a function of time *t*. The “overdrive” function is a 4 kV step function at *t* = 0 s followed by an exponential decrease to 2.5 kV in 1 s (see Supplementary Note 4). The strain is normalized to the strain at *t* = 1 s to be consistent with the results presented for the voltage step. The device response over the first 1 s is presented in Fig. 5(c), with the normalized strain in black and the corresponding strain rate in red. The device reaches 50% of the maximum strain in 0.09 ms, generating strain rate as high as 870 s^−1^. The response time could be even further improved by optimizing the shape of the overdrive signal and by increasing the initial voltage step.

The dynamics of the system are limited by the mechanical response time, not by electrical charging time constants. Modeling the DEA as a parallel plate capacitor and neglecting its mechanical deformation, the electrical time constant is given by *τ*_*RC*_ = *εAR*/*e*, where *ε* is the dielectric permittivity of the membrane, *A* the active are of the DEA, *R* the resistance of the DEA electrodes and *e* the initial thickness of the membrane. Taking an active area of 25 mm^2^, a relative dielectric permittivity of *ε*_*r*_ = 2.8, and a membrane thickness of *e* = 30 μm, the device electrical time constant is *τ*_*RC*_ ≈ 4 μs. The charging time of the device was however dominated by the slew rate of the high-voltage amplifier and by the parasitic capacitance of the wiring, leading to an electrical time constant equal to *τ*_*RC*_ ≈ 40 μs. The electrical time constant is thus more than one order of magnitude shorter than the 500 μs measured to reach 50% of the final strain when driving the device with a voltage step, which corresponds to a mechanical time constant of *τ*_*Mech*_ ≈ 700 μs. When actuated with a voltage step, the system is thus not limited by its electrical time constant, but rather by its mechanical response.

We present in Supplementary Video S1 a slow motion video of the DEA cell stretcher reproducing the extreme mechanical environment of a violent impact. It shows the device reaching the set strain in less than 1 ms after actuation (3 kV voltage step), exposing cells to strain rates of ~500 s^−1^.

### Live cell fluorescence imaging

**Figure 6:**
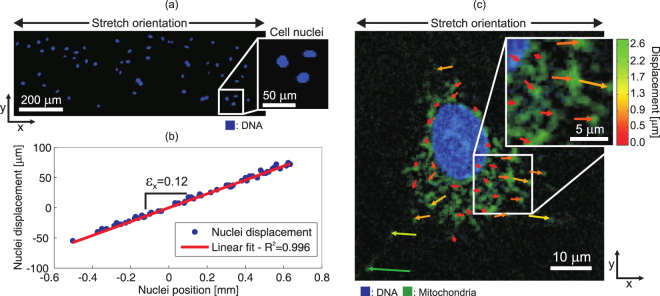
The optical transparency and compact design of the device enable live cell imaging during stretching. Lung cells A459 were cultured on the device and stained to show DNA in blue and mitochondria in green. Microscope images are in the strained configuration. (**a**) At 10x magnification the field of view is sufficiently large to image the entire electrode gap. The strain generated along the actuation orientation *ε*_*x*_ = 0.12 is measured by tracking the displacement of every nucleus. (**b**) At 40x magnification it is possible to image intracellular content such as mitochondria, and track its displacement after stretching as presented by the color- and length-coded arrows. The arrowheads of the displacement vectors point at the final positions of the areas that were tracked. See Supplementary Video S2 for animated fluorescence images of cells being stretched.

The device’s optical transparency and compact design make it possible to image cells during stretching as presented in Fig. 6. Human lung carcinoma A549 cells were cultured on the device, which was then connected to a high-voltage power supply using a custom made holder, and transferred under a microscope. Once mounted in the holder shown in Fig. 1, the complete system is only 1.2 cm thick and 8.5 cm by 7 cm wide. The cells are separated from the bottom glass coverslip by less than 300 μm, making it possible to use standard microscope objectives with up to 40x magnification. Higher magnification is possible by using long working distance (LWD) objectives. An image acquired at 60x is presented in Supplementary Note 7.

Live cell fluorescent dyes were used to stain the DNA and mitochondria in blue and green respectively. Figure 6(a) shows an image of stretched cells captured at 10x magnification and cropped to the dimension of the electrode gap. Each blue dot corresponds to a cell nucleus as this is where most of the DNA is located. The x-coordinates of each nucleus were recorded (using the Manual Tracking tool of ImageJ) at 0 V and then with 3 kV applied on the vertical electrodes to generate uniaxial tensile strain (See Supplementary Video S2 for animated fluorescence images). Figure 6(b) shows the measured nuclei displacements as a function of the nuclei positions, demonstrating that the strain generated by the DEA is transferred to the cell monolayer. A linear regression is used to fit the results and calculate the corresponding strain *ε*_*x*_ = 0.12. Imaging at low magnification is useful to study entire cell populations and to monitor functions such as migration and proliferation, whereas imaging at higher magnification allows investigating how the sub-cellular structure is affected by mechanical stimulation. Figure 6(c) shows an image of a stretched cell captured at a 40x magnification where the nucleus and the mitochondria are visible in blue and green respectively. The x- and y- coordinates of several points were recorded (using the Manual Tracking tool of ImageJ) at 0 V and then with 3 kV applied on the vertical electrodes to generate uniaxial tensile strain (See Supplementary Video S2 for animated fluorescence images). The measured displacements are shown using length- and color- coded arrows, where the arrowheads point at the final positions of the areas that were tracked. The y-component (perpendicular to stretch) observed on several displacement vectors is due to the fact that the membrane stretch is not directly transferred to intracellular elements such as mitochondria. Additional fluorescence images acquired on the device at 40x and 60x magnification are presented in Supplementary Note 7. We also present in Supplementary Video S2 a series of short animations which display fluorescence images at the rest and actuated states, clearly showing the cell deformation upon actuation of the DEA cell stretcher.

## Conclusion

We presented a DEA cell stretcher for mechanical stimulation of cell monolayers, generating from −12% to +38% uniaxial strain with a sub-millisecond response time. The ultra-fast response time, combined with the possibility to apply tensile and compressive stress make it possible to accurately reproduce the complex mechanical strain-time profile experienced by cells *in vivo*. Generating strain rates as high as 870 s^−1^, the device can reproduce extreme mechanical environments such as those experienced after blows to the head, leading to traumatic brain injuries. The DEA-based device has a compact design and its excellent optical transparency enable fluorescent live cell imaging during stretching. The device’s unique capabilities enable new experiments that can foster progress in the emerging field of mechanobiology. This new technology could provide an insightful look into important questions such as the role of mechanics in actin stress fiber kinetics, and the role of strain-rate in axonal injury. Future work will focus on the development of a compact array of individually addressable DEA cell stretchers. Controlled by a single high-voltage power supply, the integrated array of DEA cell stretchers will provide a compact solution for high-throughput cell stretching experiments.

## Materials and Methods

### Cell stretcher fabrication process

The device consists of a 30 μm-thick silicone elastomer (Sylgard 186, Dow Corning) membrane stretched by *λ*_*H*_ = 2.7 on one axis and by *λ*_*L*_ = 1.2 on the perpendicular axis, as shown in Fig. 3(a). The membrane is fixed between rigid Poly(methyl methacrylate) (PMMA) supports and carbon-elastomer composite electrodes^50^ are patterned on both sides to create a DEA. A PMMA ring is bonded on the membrane, creating a well where cells can be cultured and which can contain approximately 2 ml of growth medium. A thinner PMMA ring is bonded on the bottom side of the membrane, which is then filled with a 100 μm-thick layer of oil, and encapsulated using a 150 μm-thick glass coverslip. With this compact assembly, cells are separated from the optics by less than 300 μm when imaging from below the device. We reported a detailed version of the fabrication process^46^.

### Strain measurements

The actuation strain was calculated from the width *e* and length *g* of the gap that separates the two vertical electrodes as defined in Fig. 3(b). The values measured at rest (*e*, *g*) and in the actuated state (*e*′, *g*′) were used to calculate the average strain along the low prestretch orientation *ε*_*x*_ = *e*′/*e* − 1 and high prestretch orientation *ε*_*y*_ = *g*′/*g* − 1. The local strain distribution (Fig. 4) was mapped by processing pictures of the cell culture at rest and in the actuated state using a digital image correlation tool^53^. The device was actuated using a waveform generator connected to a high-voltage power amplifier (609E-6, TREK) and pictures of the actuator were taken using a high-speed camera (Phantom V210, Vision Research).

### Cell preparation protocol

For the strain measurements we worked with human bronchial smooth muscle primary cells (CC-2576 from Lonza). The samples were sterilized with ethanol 70%, rinsed in sterilized DI water and coated with fibronectin at a concentration of 50 μg ml^−1^. The cells were seeded at density of 5 × 10^5^ cells/mm^2^ and incubated for 24 h. For the fluorescence imaging we worked with human lung carcinoma A549 cells. The samples were sterilized with ethanol 70%, rinsed in sterilized DI water and coated with fibronectin at a concentration of 12 μg ml^−1^. The cells were seeded at a density of 0.5 × 10^5^ cells/mm^2^ and grown to reach 80% confluence.

### Fluorescence imaging protocol

For fluorescence imaging we worked with human lung carcinoma A549 cells. The cultured cells were exposed for 30 min to a live cell-staining solution containing mitochondrial dye MitoTracker Green FM (Invitrogen, USA), or transfected for 16 h with CellLight mitochondria-RFP (Thermo Fisher Scientific, USA). Cells were then stained with Hoechst (Invitrogen) for 10 min to visualize nuclei. A preheated Nikon A1r confocal microscope (Nikon, Tokyo, Japan) with 10x, 40x and 60x objectives was used to observe mitochondria and nuclei. The Manual tracking tool from ImageJ was used to track displacement of nuclei and mitochondria between the fluorescence images acquired at rest and actuated state.

## Electronic supplementary material

Supplementary information

Video S1

Video S2
