# Supplementary material for: An ultra-fast mechanically active cell culture substrate
Source: Sci Rep. 2018 Jul 2;8:9895. doi: 10.1038/s41598-018-27915-y (PMC6028580; doi:10.1038/s41598-018-27915-y)
Supplement: Supplementary file 1 — Supplementary information [file 41598_2018_27915_MOESM1_ESM.pdf]

Supplementary information -

An ultra-fast mechanically active cell culture substrate

Alexandre Poulin[1], Matthias Imboden[1], Francesca Sorba[1,2], Serge Grazioli[3,4], Cristina Martin-Olmos[2], Samuel Rosset[1,5], and Herbert Shea[1,]*

[1]Institute of Microengineering, Ecole Polytechnique Fédérale de Lausanne, Neuchâtel, Switzerland

[2]Swiss Center for Electronics and Microtechnologies (CSEM), Neuchâtel, Switzerland

[3]Division of Neonatology and Pediatric Intensive Care Medicine, University Hospital of Geneva, Geneva, Switzerland

[4]Department of Microbiology and Molecular Medicine, Faculty of Medicine, University of Geneva, Geneva, Switzerland

[5]Bioengineering Institute, University of Auckland, Auckland, New Zealand

*herbert.shea@epfl.ch

Supplementary Note 1 – DEA cell stretcher design and control

The DEA cell stretcher design shown in Fig. S1(a)-(b) maximises the strain uniformity in the central transparent gap. The vertical electrodes generate tensile strain whereas the horizontal electrodes generate compressive strain. The high aspect ratio (*e*>*g*) of the gap separating the vertical electrodes minimizes the border effects (necking in the strain profile), while the narrow *w* feedlines minimize the actuation along the y-axis and provide a more uniaxial deformation (i.e. it maximizes $\varepsilon_x/\varepsilon_y$). The horizontal electrodes are designed to expand along their length and compress the central transparent gap of dimension *g* x *e*. The width *w* of the horizontal electrodes has to be larger than the gap *w*>*g* to provide a uniform strain profile, but significantly smaller than the membrane width to maximise the actuation strain[1]. It is also important to keep transparent passive regions in the membrane to be used as control areas where cells are not exposed to mechanical strain.

The electrical contacts between the DEA cell stretcher and the voltage power supply are achieved using a custom made holder presented in Fig. S1(c). To connect the device, each metallic pad is coated with a drop of electrolyte gel and the device is assembled on top of the holder. The holder is then connected to a high-voltage power supply[2] as presented in Fig. S1(d). The device can be controlled with a single bi-polar power supply and a bridge rectifier, connecting the positive and negative output of the rectifier to the tensile and compressive electrodes respectively. In this configuration, the actuation mode of the DEA cell stretcher is controlled by the polarity of the driving signal.

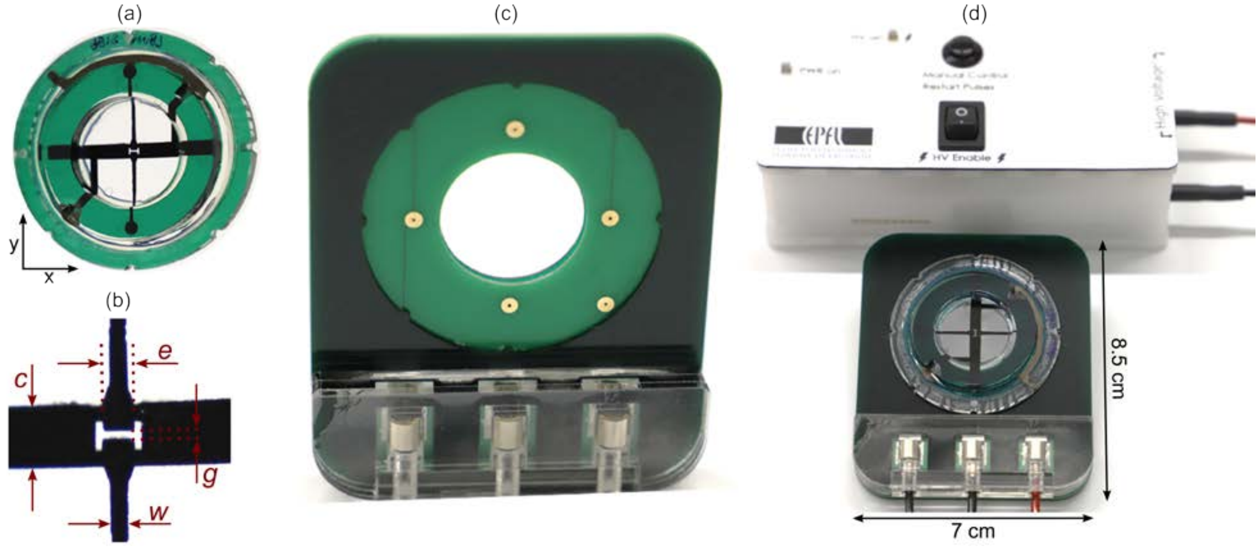

Figure S1: *(a) Picture of the DEA cell stretcher. (b) Close-up view of the electrodes geometry. (c) Custom made holder we developed to make electrical contacts between the device and its high-voltage power supply. (d) Picture of the device assembled on its holder and connected to a compact high-voltage power supply*[2].

Supplementary Note 2 – DEA cell stretcher fabrication process

The main fabrication steps of the DEA cell stretcher are presented in Fig. S2. In addition to the information provided here, the fabrication process of a DEA cell stretcher[3], as well as protocols for DEA materials preparation and processing[4] have been reported in prior work.

(a) The cell stretcher fabrication starts with a silicone (Sylgard 186, Dow Corning) membrane which is stretched biaxially by λ_L=1.2 in one axis, and λ_H=2.7 in the perpendicular axis. The prestretched membrane is then fixed between two rigid Poly(methyl methacrylate) (PMMA) frames using silicone adhesive (ARclear, Adhesive Research) and silicone RTV (Silpuran 4200, Wacker).

(b) Stretchable electrodes made from a carbon-elastomer composite are pad-printed on the bottom side of the membrane and heat cured, forming the high-voltage electrodes of the DEA.

(c) Stretchable electrodes made from a carbon-elastomer composite are pad-printed on the top side of the membrane and heat cured, forming the ground electrodes of the DEA.

(d) Rigid PMMA frames are assembled on either sides of the membrane using a biocompatible silicone (Silbione LSR 4305, Bluestar Silicones) for the top frame, and silicone adhesive for the bottom frame. The top frame creates a culture well which can hold approximately 1 ml of growth medium.

(e) The inner PMMA frames have openings that give access to the underlying DEA electrodes. Those openings are filled with conductive silicone RTV (SS-27S, Silicones Solutions) to provide reliable electrical contacts between the DEA electrodes and external electronics.

(f) The bottom well is covered with a glass coverslip, hence creating a microfluidic reservoir which is then filled with vegetable oil and sealed using silicone RTV. The oil immersion provides a barrier between the cell culture and the external environment, otherwise only separated by a 30 micron thick silicone membrane. The immersion also prevents optical degradation of the silicone membrane (i.e. formation of micro cracks at its surface) over periodic actuation.

Once the fabrication is completed, the culture well is filled with growth medium and cells are cultured directly on top of the DEA. The compact transparent device can be easily mounted on top of an inverted microscope for live cell imaging. Cells are separated from the glass coverslip by approximately 0.3 mm, which gives a lower limit for the working distance of microscope objectives compatible with the system.

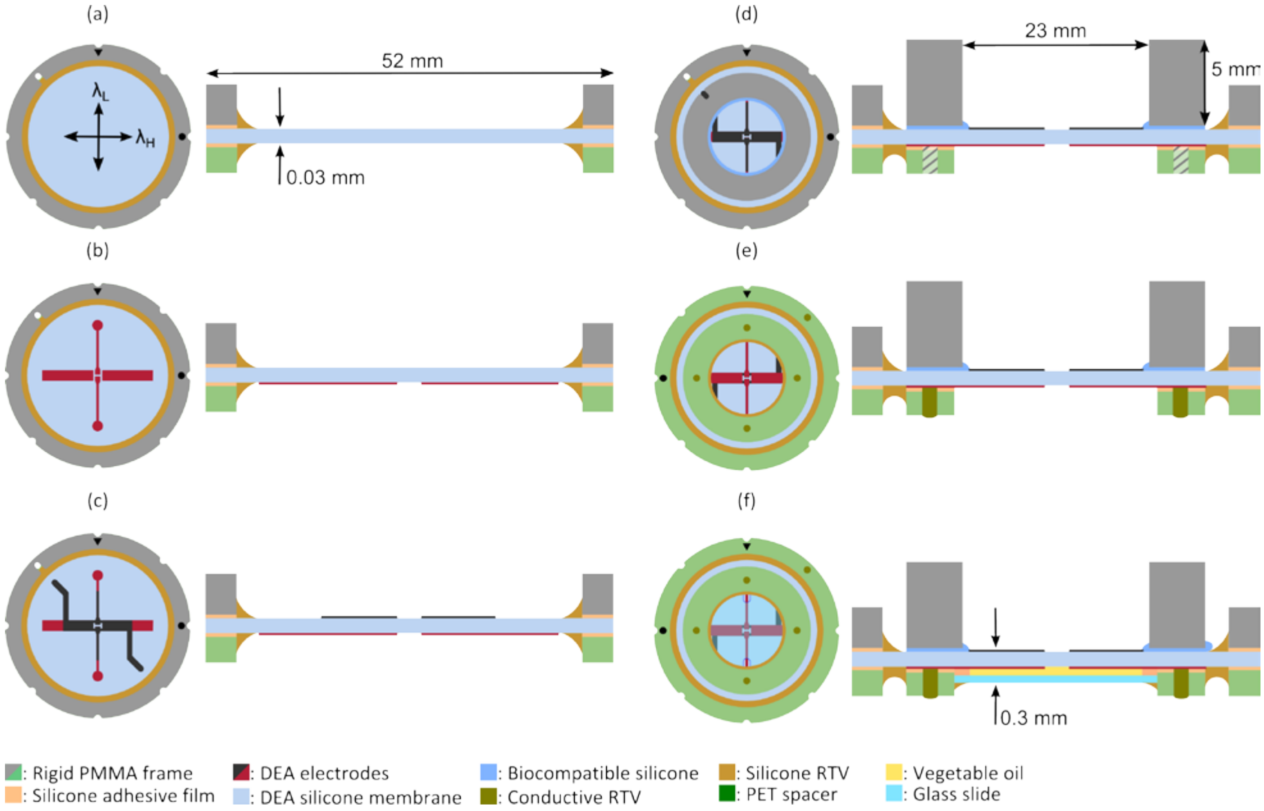

Figure S2: *Fabrication process of the DEA cell stretcher. (a) A silicone membrane is prestretched and fixed on a rigid frame. (b)-(c) Stretchable electrodes are pad-printed on both sides of the membrane to create the DEA. The top and bottom electrodes are respectively the high-voltage and ground electrodes. (d) Two rigid frames are assembled on the device, creating a culture well on top of the device which can hold approximately 1 ml of growth medium. (d) Conductive silicone rubber is used to create a reliable electrical contact between the DEA electrodes and the external electronics. (f) The bottom well is covered with a glass coverslip, hence creating a microfluidic reservoir which is then filled with vegetable oil and sealed.*

Supplementary Note 3 – Voltage step response

The device response was characterized for voltage steps of increasing amplitude and the results are presented in Fig. S3. The strain is normalized to the strain reached 1 s after the voltage step is applied. The results show that the response time of the DEA cell stretcher is independent of the driving voltage and actuation strain. A voltage-independent transfer function can therefore be used to describe the device and calculate the exact driving signal required to precisely mimic any time-varying strain profile. Future work will investigate this approach and evaluate its reliability.

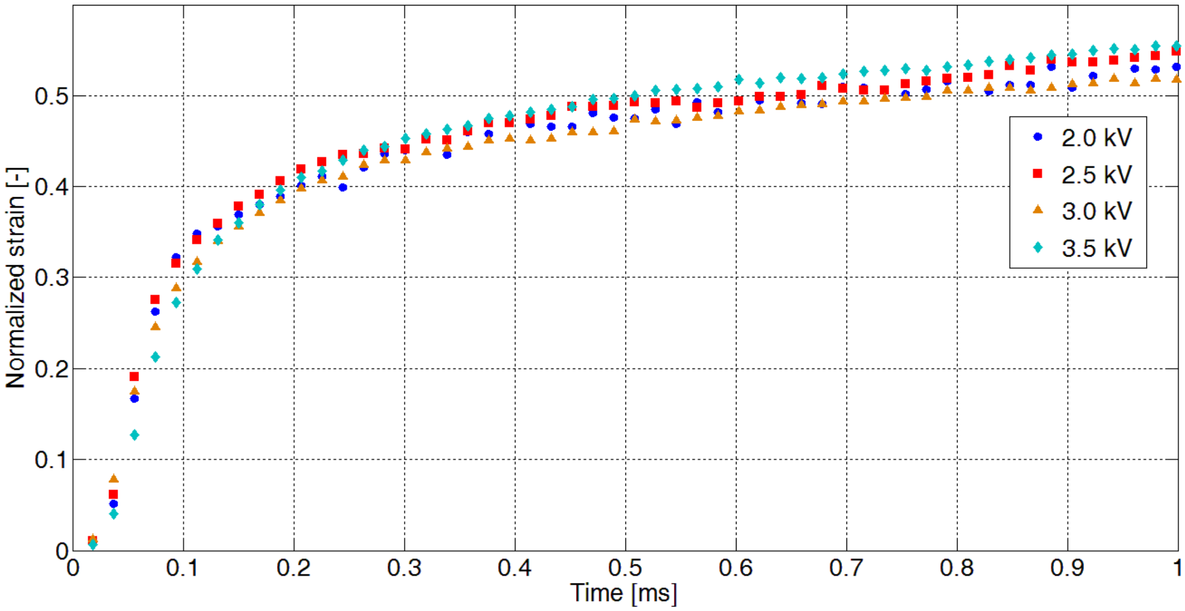

Figure S3: *Normalized actuation strain of the DEA cell stretcher as a function of time. The device was actuated using voltage steps of amplitude ranging from 2.0 kV to 3.5 kV. The measurements were made on the same device and show that the response time of the cell stretcher is independent of the driving voltage or the actuation strain.*

Supplementary Note 4 – Overdrive function

The response of the DEA cell stretcher to an overdrive function is discussed in the main manuscript. The function we used is describes by equation (S1) where *V* is the driving voltage in volt and *t* is the time in millisecond.

$$V = \begin{cases} 0\,, & -500 \le t < 0 \\ 500 \cdot \left(e^{-t/100} + e^{-100t^2} + e^{-10t^3}\right) + 2500, & 0 \le t \le 1500 \end{cases} \tag{S1}$$

The shape of the overdrive function is presented in Fig. S4. At 0 ms the voltage instantly ramps from 0 V to 4 kV (due to the finite resolution of the waveform generator the voltage step takes 0.1 ms) after which it exponentially decreases to 2.5 kV over the following 1,000 ms. This empirical function was obtained by trial and error and could be further improved.

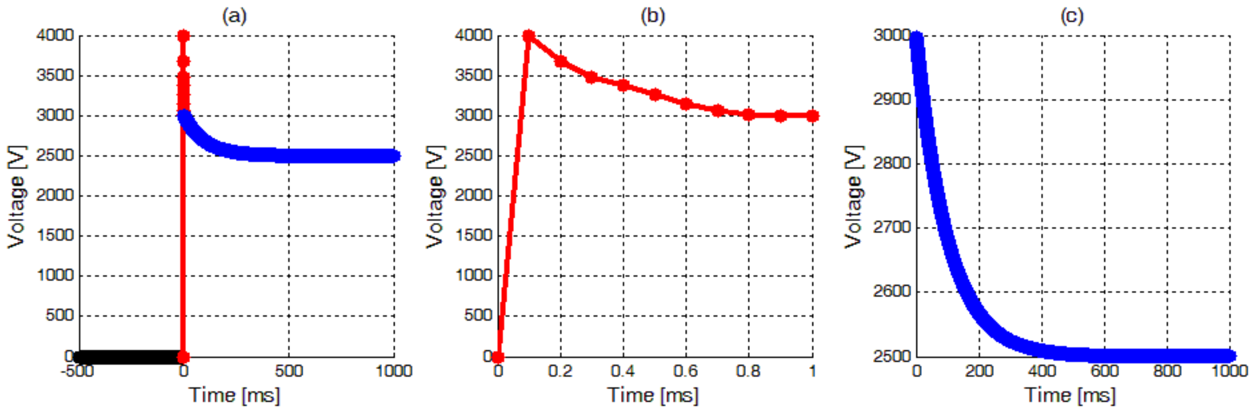

Figure S4: *(a) Graphical representation of the overdrive function discussed in the main manuscript. The voltage is initially set to 0 V (black line). At time t=0 ms, the voltage jumps from 0 V to 4 kV, after which it exponentially decrease to 2.5 kV in 1 s. The red segment corresponds to the first 1 ms of the overdrive function after the voltage step, whereas the blue segment corresponds to the next 999 ms. (b) Graphical representation of the overdrive function in the interval of 0-1 ms after the voltage step. (c) Graphical representation of the overdrive function in the interval of 1-1,000 ms after the voltage step. Using this overdrive signal, the desired strain was reached within less than 1 ms.*

Supplementary Note 5 – Frequency response

The frequency response of the DEA cell stretcher was characterized in tensile mode. The actuation strain was measured at different frequencies using a 3 kV sinusoidal signal and the results are presented in Fig. S5(a). As the frequency increases, the device doesn't have time to fully expand or relax between cycles and the actuation strain decreases. As discussed in the main manuscript, the system is limited by its mechanical time constant and not by its electrical time constant. We calculated the strain amplitude $\Delta\varepsilon=\varepsilon_{max}-\varepsilon_{min}$ as the difference between the maximum ε_{max} and minimum ε_{min} strain values, and normalized it to the strain amplitude measured at 0.1 Hz. The normalized strain amplitude is reported in Fig. S5(b) as a function of frequency for different driving voltages. The results show that the cut-off frequency varies with the driving voltage and actuation strain, and is equal to 800 Hz at 3 kV.

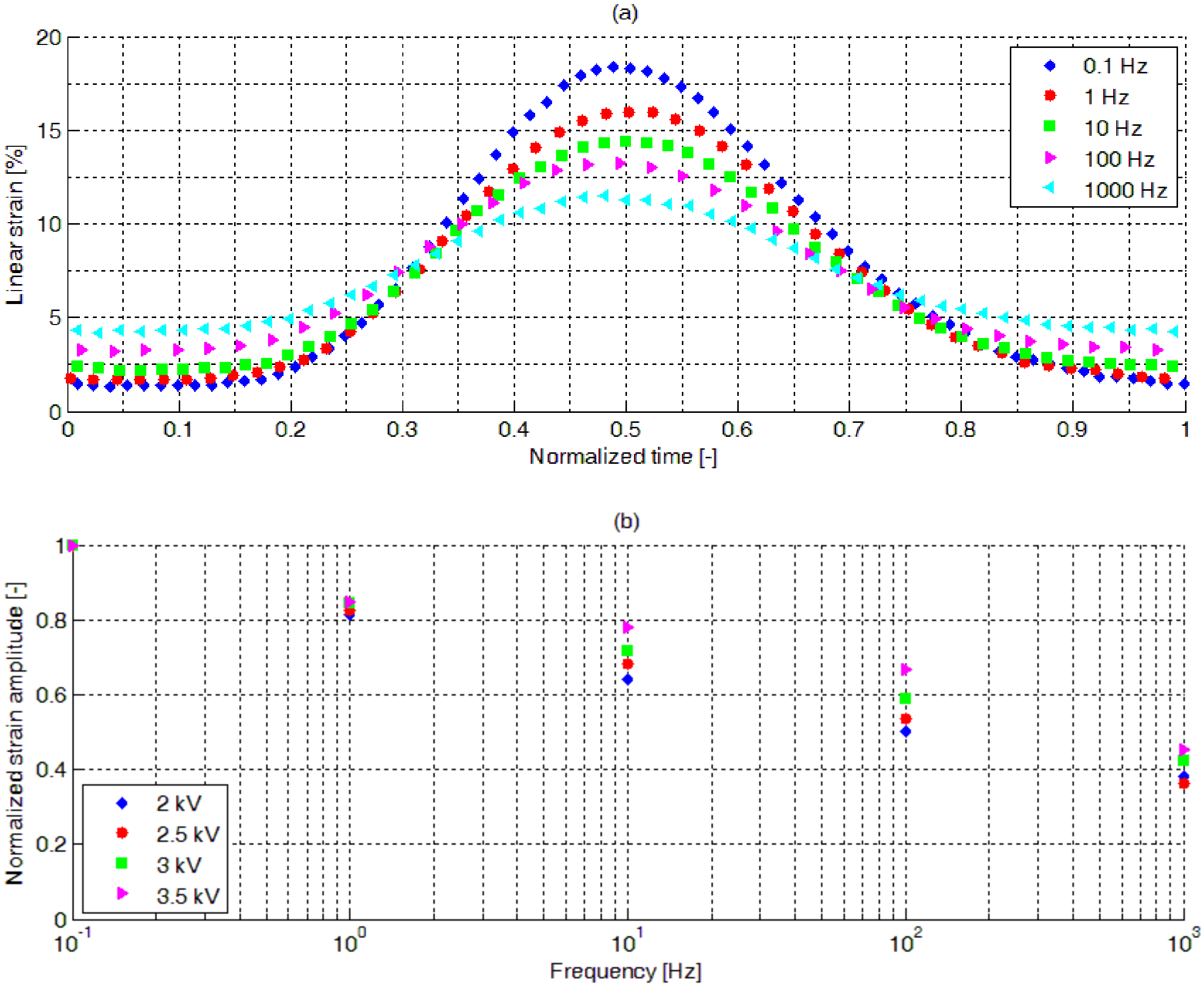

Figure S5: *(a) Actuation strain measured at different frequencies using 3 kV sinusoidal driving signals. The time is normalized to the period of the driving signal. (b) Normalized strain amplitude as a function of frequency for different driving voltages. The normalized strain amplitude* $\Delta\varepsilon=\varepsilon_{max}-\varepsilon_{min}$ *is the difference between the maximum* ε_{max} *and minimum* ε_{min} *strain measured, normalized to the strain amplitude measured at 0.1 Hz.*

Supplementary Note 6 – Lifetime and stability

The lifetime and stability of the DEA cell stretcher was characterized in culture conditions. The culture chamber was filled with growth medium and the device was actuated using a 1 Hz square wave signal with a 50 % duty cycle. Every 10'000 cycles, the strain was measured at rest (0 V) and in the actuated state (4 kV), waiting 1 s between applying the voltage and measuring the strain. The results obtained over 80,000 actuations cycles are reported in Fig. S6 as a function of time. The experiment was stopped after 80,000 cycles due to a failure of the DEA by dielectric breakdown. The strains at rest and in the actuated state show a small drift over the first 10,000 cycles, after which the actuation strain remains very stable over the next 70,000 cycles. The strain drift can be due to a complex combination of stress relaxation, viscoelasticity, and plastic deformation of the membrane and the electrodes.[3]

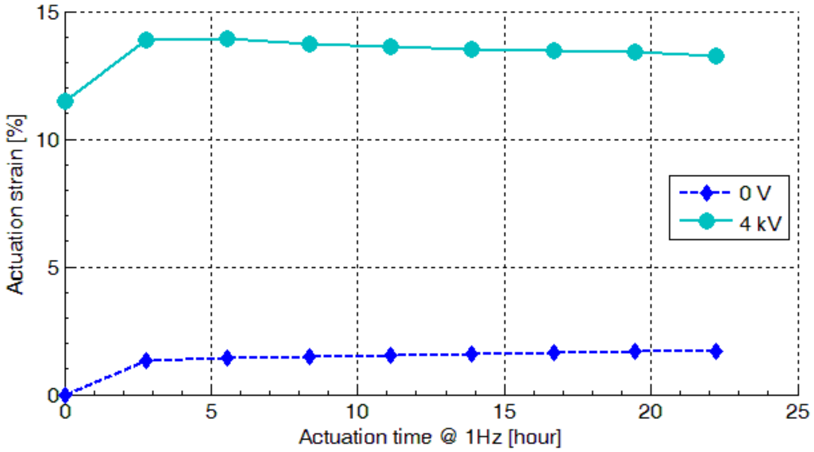

Figure S6: *Actuation strain of the DEA cell stretcher at rest (0V) and in the actuated state (4 kV) over periodic actuation. The device was actuated using a 1 Hz square wave signal with a 50 % duty cycle. The experiment was stopped after more than 20 hours of operation and 80'000 actuation cycles due to the DEA failure by dielectric breakdown. The device exhibit a small 1-2 % strain drift over the first 10'000 cycles, after which the actuation strain remains very stable.*

Supplementary note 7 – Live cell imaging

The optical transparency and compact design of the DEA cell stretcher enables live cell imaging. Inverted microscope objectives can be brought 300 μm from the cultured cells, limited by the thickness of the DEA membrane and the oil backing. Standard objectives can be used at up to 40x magnification as seen in Fig. S7(a) which shows human lung carcinoma A549 cells imaged at 40x magnification (DNA and mitochondria are seen in blue and green respectively). Higher magnification can be achieved using long working distance objectives as seen in Fig. S7(b) which shows human lung carcinoma A549 cells imaged at 60x magnification (DNA and mitochondria are seen in blue and red respectively).

Between the rest and actuated (10 % strain) states, the cell monolayer moves by approximately 30 μm out-of-plane. It is therefore necessary to refocus the microscope when working at high magnification. The amplitude of the displacement increases with the driving voltage and the actuation strain. For a 10 % actuation strain on a 30 μm -thick membrane, the thickness compression contributes to the out-of-plane motion by only 1.5 μm. The motion is induced mainly by electrostatic forces generated between the high-voltage electrodes and the microscope objective, effectively pulling the membrane towards the objective when the DEA is actuated. This effect can be suppressed by changing our two electrodes configuration to a three electrodes configuration where a central high-voltage electrode is bounded on both sides by ground electrodes.

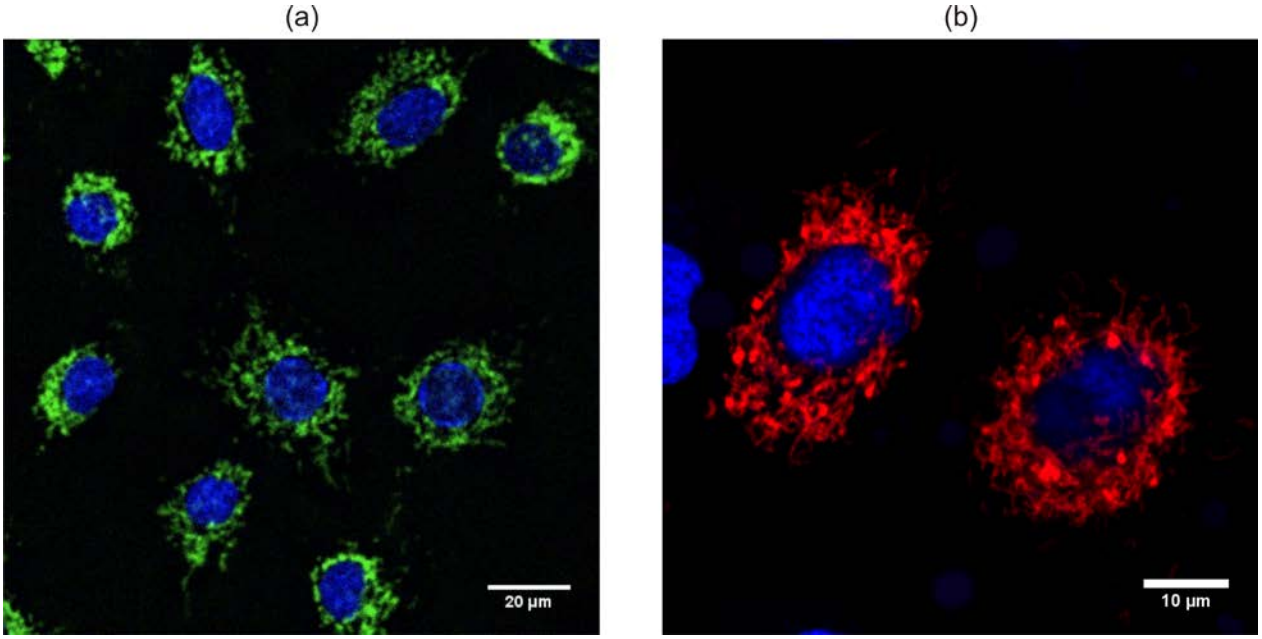

Figure S7: *Fluorescence imaging of human lung carcinoma A549 cells on the DEA cell stretcher. (a) Cells were imaged at 40x magnification and stained with MitoTracker Green FM (Invitrogen, USA) to show mitochondria in green and Hoechst (Invitrogen, USA) to show DNA in blue. (b) Cells were stained with CellLight mitochondria-RFP (Thermo Fisher Scientific, USA) to show mitochondria in red and Hoechst (Invitrogen, USA) to show DNA in blue, and imaged with a Plan Apochromat Lambda 60x/1.4 oil immersion objective lens.*

Supplementary Videos legends

Supplementary Video S1

Light microscopy videos demonstrating the device's ultra-fast response time, its capability to reproduce complex strain profiles, and compatiblity with high magnification microscope objectives. The first clip shows the device's response to a 3 kV voltage step. The video was recorded using a high-speed camera and it is slowed down by 1,000x in order to see the sub-millisecond response time of the system. The second clip shows the device generating a complex strain profile that reproduces the mechanical environment of the myocard. The last clip shows a real time video of cells being stretch and imaged at 40x magnification.

Supplementary Video S2

Animations showing fluorescence images of cells on the DEA cell stretcher at the rest and actuated states. Human lung carcinoma A549 cells were stained to see DNA in blue (Hechst, Invitrogen) and mitochondria in green (MitoTracker Green FM, Invitrogen), and imaged using a Nikon A1r confocal microscope. The first animation displays images acquired at 10x magnification, showing the displacement of a small cell population's nuclei upon actuation. The second and third animations display images acquired at 40x magnification, showing the deformation of single cells and their intracellular content upon actuation.

References

1. Rosset, S., Araromi, O. A. & Shea, H. R. Maximizing the displacement of compact planar dielectric elastomer actuators. *Extrem. Mech. Lett.* 3, 72–81 (2015).

2. Rosset, S., Schlatter, S. & Shea, H. Project Peta-pico-Voltron. http://petapicovoltron.com (2017)

3. Poulin, A., Shea, H. R. & Rosset, S. Fabrication and characterization of silicone-based dielectric elastomer actuators for mechanical stimulation of living cells. In *Proc. SPIE 10594, Electroactive Polymer Actuators and Devices (EAPAD) XX*, 105940V (2018)

4. Rosset, S., Araromi, O. A., Schlatter, S. & Shea, H. R. Fabrication Process of Silicone-based Dielectric Elastomer Actuators. *J. Vis. Exp.* 108, e53423; 10.3791/53423 (2016).
